# Supplementary material for: Detecting recent selective sweeps while controlling for mutation rate and background selection
Source: Mol Ecol. 2015 Sep 7;25(1):142–56. doi: 10.1111/mec.13351 (PMC5082542; doi:10.1111/mec.13351)
Supplement: Supplementary file 1 — Appendix S1. Command line for msms (Ewing & Hermisson 2010) simulations. Fig. S1 Power of the CLR tests for data with different levels of divergence from the outgroup. Fig. S2 Boxplot of the distribution of the number of segregating sites for the 18 different bottleneck scenarios, calculated for the simulated 100 kb sequence and 200 replications each. Fig. S3 Distribution of Tajima's D for the 18 bottleneck scenarios, calculated for the simulated 100 kb sequence and 200 replications each. Fig. S4 FPR under both population bottleneck and reduced mutation rate. Fig. S5 Reduction in diversity due to the effect of background selection (B‐‐‐value map) calculated from forward simulations with SFS_CODE under a constant size model (see Materials and Methods), and under a bottleneck model with parameters for European humans from Lohmueller et al. (2011a,b). Fig. S6 a) The observed proportion of false positives in case of simulations with background selection plotted against the nominal false positive rate (significance level). The nominal false positive rate is estimated from neutral simulations without background selection. b) The power of detecting a recently fixed selective sweep with Nes = 200 as a function of the proportion of false positives. Fig. S7 FPR and power under both background selection and a population bottleneck. Fig. S8 Examples from the human genome scan, running both the standard version of CLR using only polymorphic sites (CLR1), and our new version including fixed differences and the B‐value map (CLR2). [file MEC-25-142-s001.pdf]

Supplement

Supplemental Text

Appendix S1: Command line for msms (Ewing & Hermisson 2010) simulations.

msms -ms 31 1 -t $theta -r $recombinationRate 1000 -I 2 1 30 0 -n 2 1 -en $onset 2 $strength -en ($onset + $duration) 2 1 -ej $splitTime 2 1 –N 10000 –SFC –SI $sweepStart 0 5e-05 –Sc 0 2 $selectionCoeff –Sp 0.5

We set the split time (g) between ingroup and outgroup ($splitTime) to 10. The population mutation rate θ ($theta) is set to 0.005*L and the population scaled recombination rate ($recombinationRate; $2N_e r$) to 0.01*L, where L is the sequence length of 10kb. To analyze the effect of reduced mutation rate, we varied the mutation rate to be 0.1, 0.2,…,0.9 times smaller than the background mutation rate.

In simulations with selection, the selected mutation was introduced in the population at specified times, $sweepStart = 0.005, 0.01, 0.02, 0.03, 0.04, 0.08, 0.12, … , 0.6, at a frequency of $1/(2N_e)$ with a population scaled selection coefficient of $selectionCoeff = 200 ($2N_e$). The selected mutation was lost in the initial stochastic phase in most cases, but we only kept simulations where the mutation did not get lost (-SFC option).

For the constant size simulations, $strength was set to 1, *i.e.* there was no change in size over time. For the bottleneck simulations, we varied $onset (0.002, 0.02 and 0.2), $strength (0.05, 0.1 and 0.5) and $duration (0.04 and 0.2) to get all possible combinations of those parameters. To fairly compare different bottleneck scenarios, mutation rate ($theta) was scaled depending on the bottleneck parameters so that SNP density is the same for all simulations (on average ~1850 SNPs per simulation). Recombination rate ($recombinationRate) was scaled with the same factor to keep the recombination over mutation rate ratio comparable to the constant size simulations. The average number of fixed differences to the outgroup was kept constant for all conditions by dividing split time ($splitTime) by the same scaling factor.

Supplemental Figures

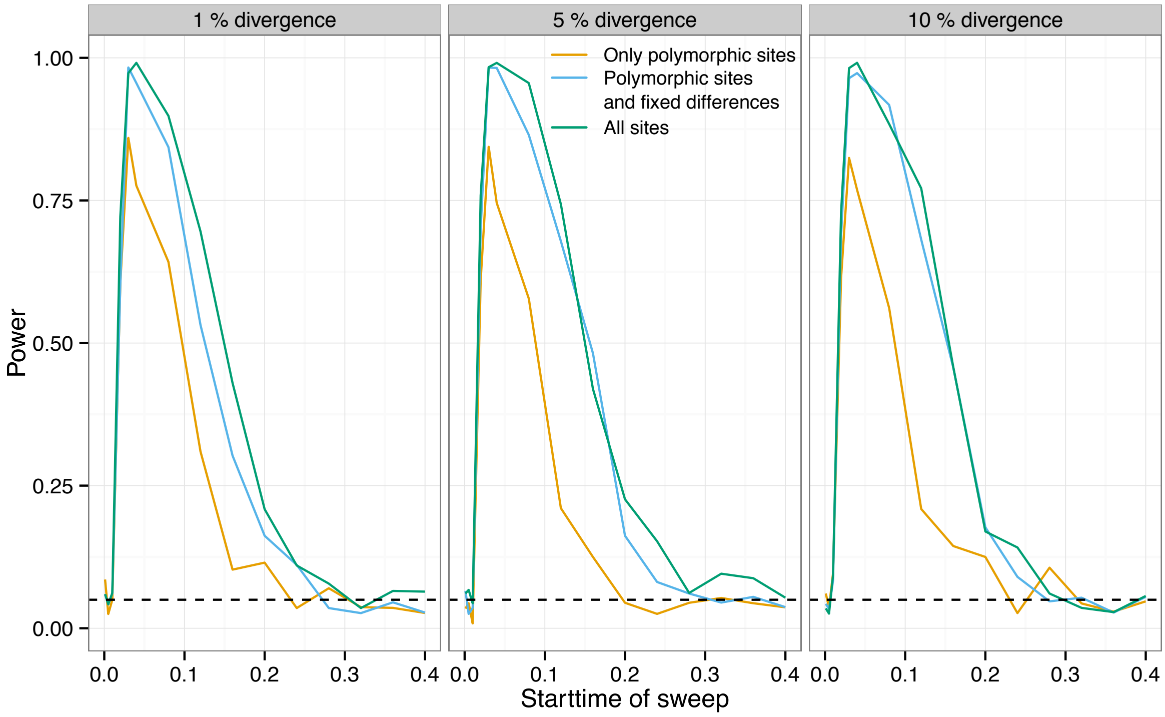

Figure S1. Power of the CLR tests for data with different levels of divergence from the outgroup. Split time between ingroup and outgroup is set such that neutral divergence is 1%, 5% and 10%. The power of the selection tests is shown as a function of the time since introduction of the beneficial mutation into the population in 2N_e generations (x-axis). The dashed line in indicates the 5% significance level assumed in the power calculations. Each 100kb simulated region is scored significant if it contains at least one significant outlier CLR at the 5% level.

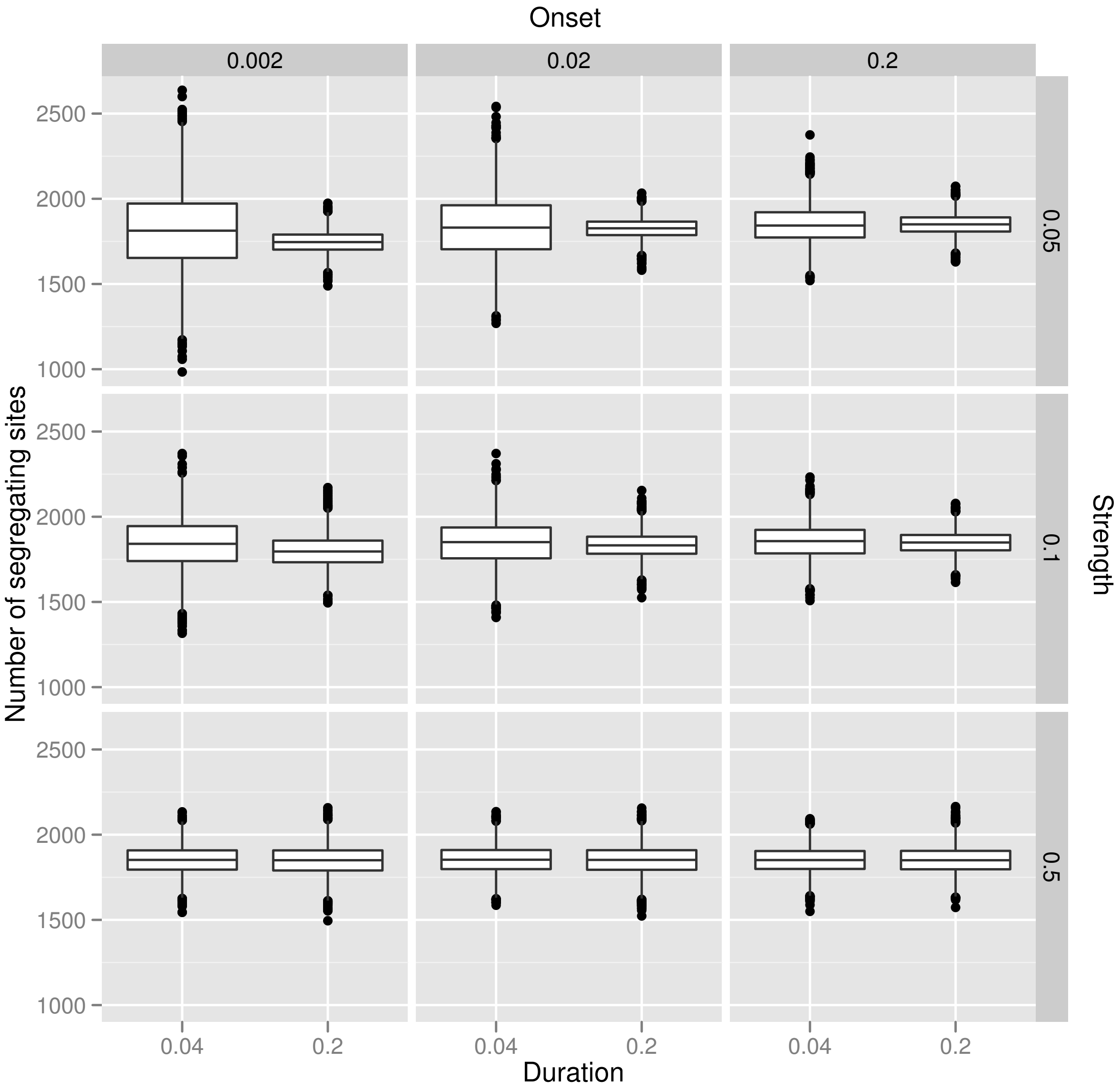

Figure S2: Boxplot of the distribution of the number of segregating sites for the 18 different bottleneck scenarios, calculated for the simulated 100kb sequence and 200 replications each. Bottleneck parameters are defined according to Figure 5.

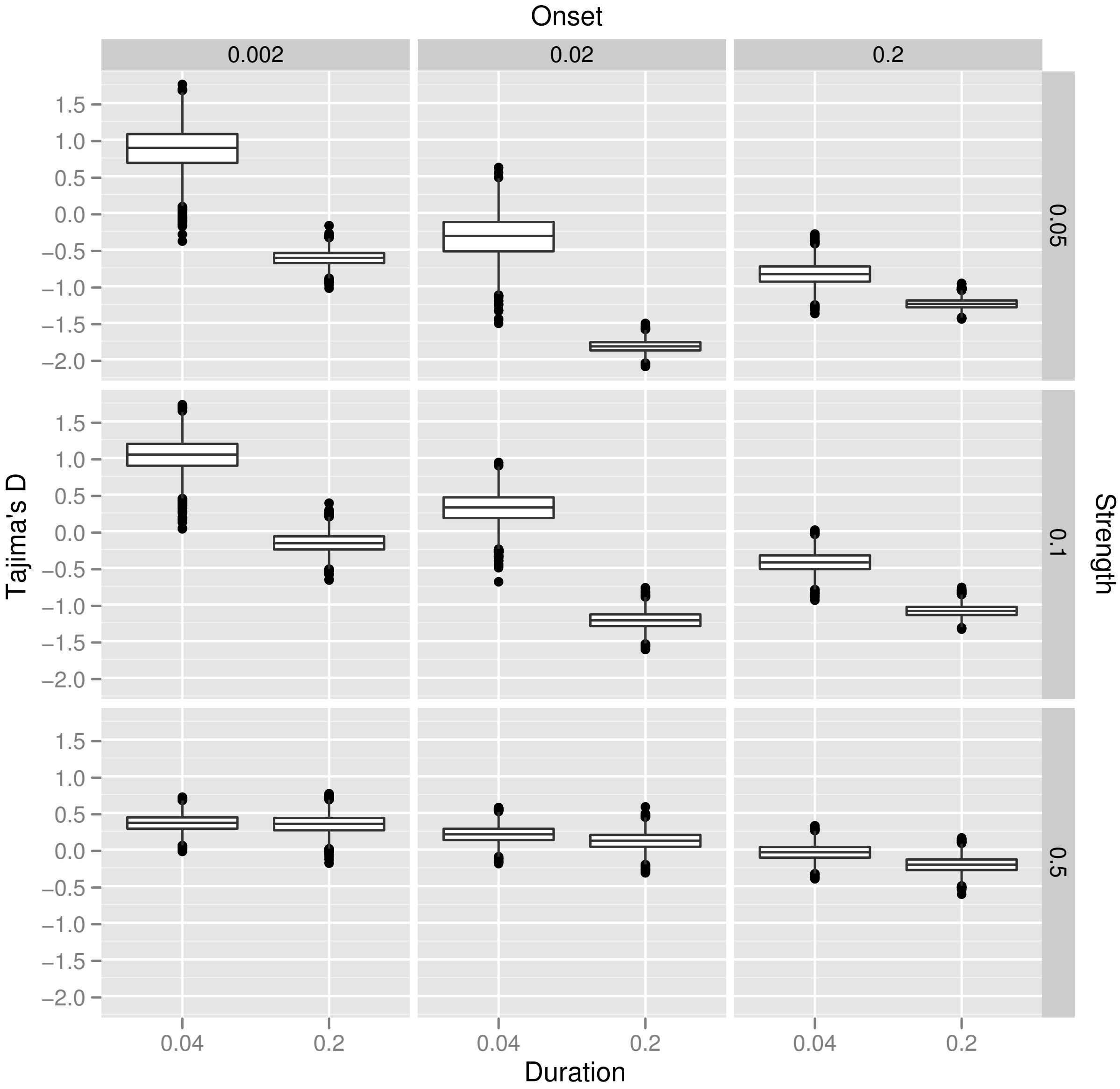

Figure S3: Distribution of Tajima's *D* for the 18 bottleneck scenarios, calculated for the simulated 100kb sequence and 200 replications each. Bottleneck parameters are defined according to Figure 5.

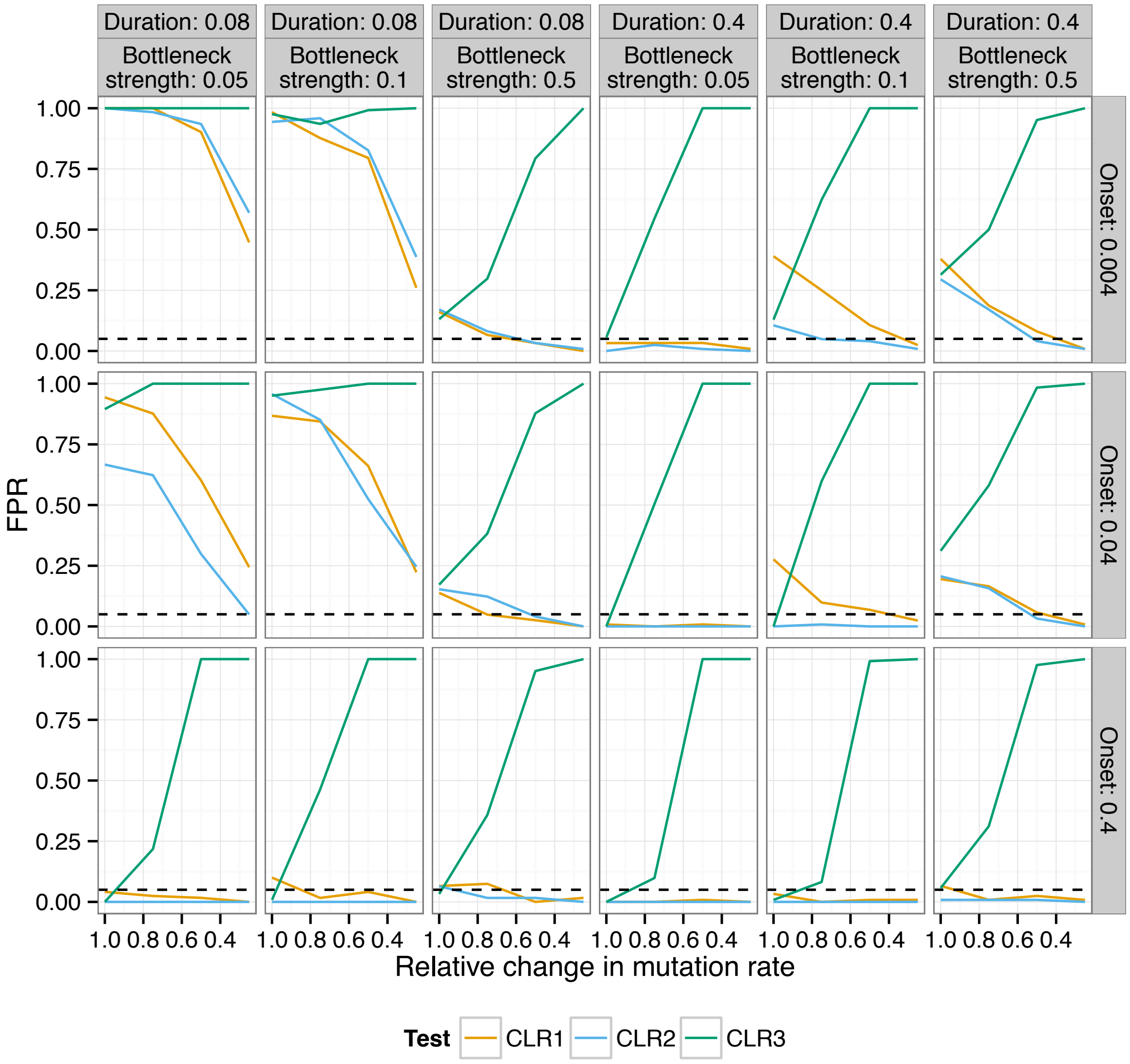

Figure S4. FPR under both population bottleneck and reduced mutation rate. 'Strength' is defined as $N_{e(b)}/N_e$ the effective population size during the bottleneck ($N_{e(b)}$) divided by the effective population size before or after the bottleneck (N_e), 'duration' is measured in number of generations divided by $2N_e$, and 'onset' is number of generations since the bottleneck started divided by $2N_e$ (see Figure 5a). FPR is based on a significance cutoff calculated from constant size simulations, similar to Figure 5b. It is shown as a function of the reduction in mutation rate of the sequence under investigation relative to the mutation rate of the sequence that is used to calculate the background SFS (similar to Figure 4). Each 100kb simulated region is scored as false positive if it contains at least one significant outlier CLR at the 5% level. Note that the background SFS is based on the (true) bottleneck model, not the constant size model.

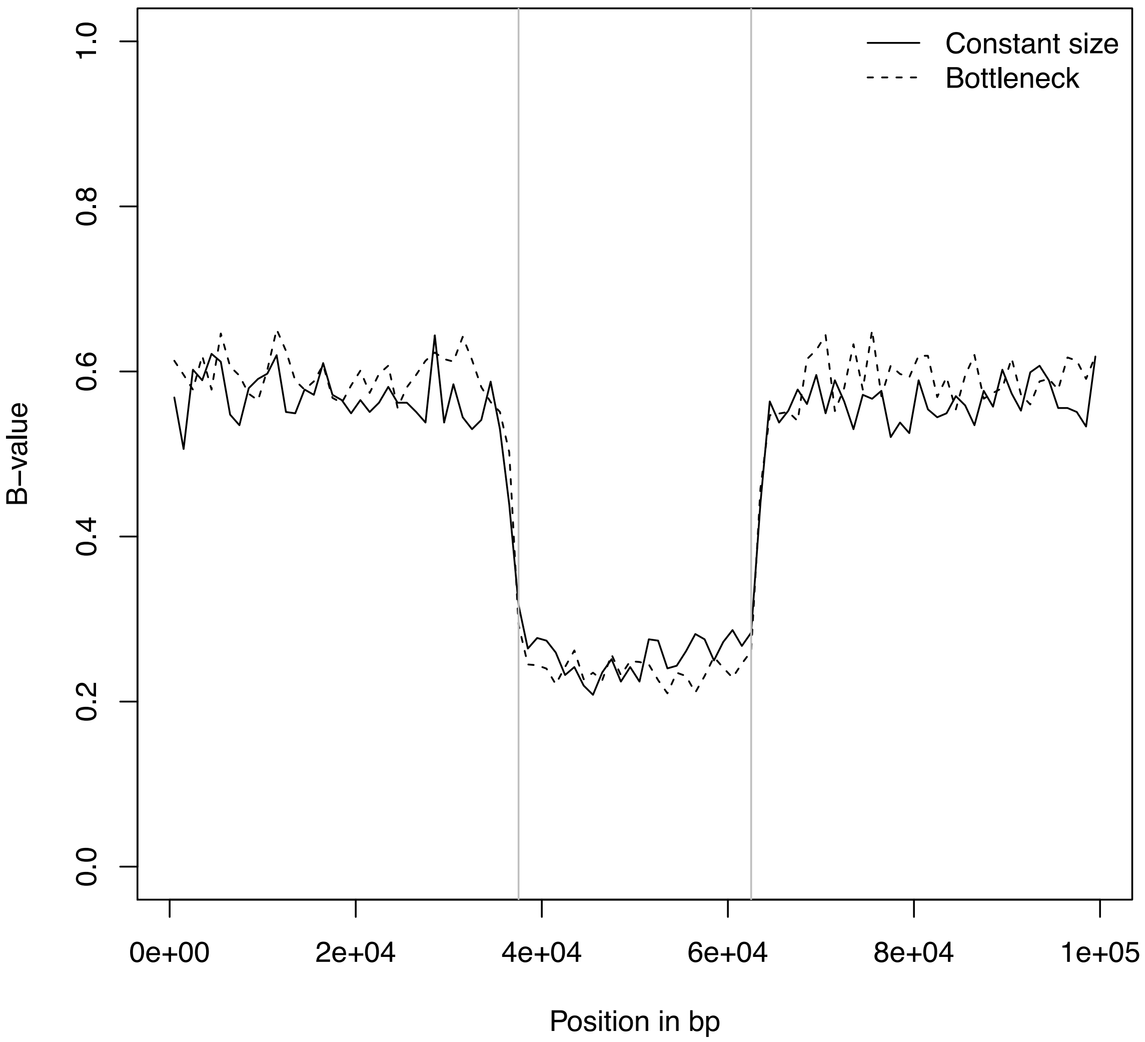

Figure S5: Reduction in diversity due to the effect of background selection (B-value map) calculated from forward simulations with SFS_CODE under a constant size model (see Materials and Methods), and under a bottleneck model with parameters for European humans from Lohmueller et al. (2011). Note that the B-value map from the bottleneck simulations is similar to the one from constant size simulations, indicating that a map that is estimated assuming a constant size population is an unbiased estimate of the map under a population bottleneck.

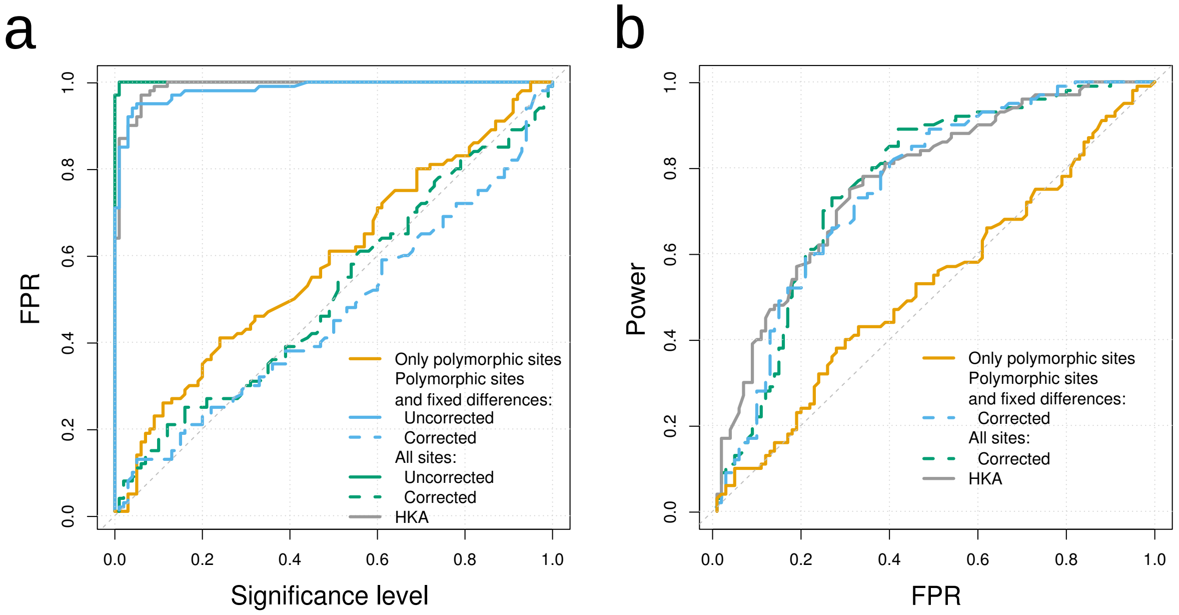

Figure S6. a) The observed proportion of false positives in case of simulations with background selection plotted against the nominal false positive rate (significance level). The nominal false positive rate is estimated from neutral simulations without background selection. b) The power of detecting a recently fixed selective sweep with $N_e s = 200$ as a function of the proportion of false positives.

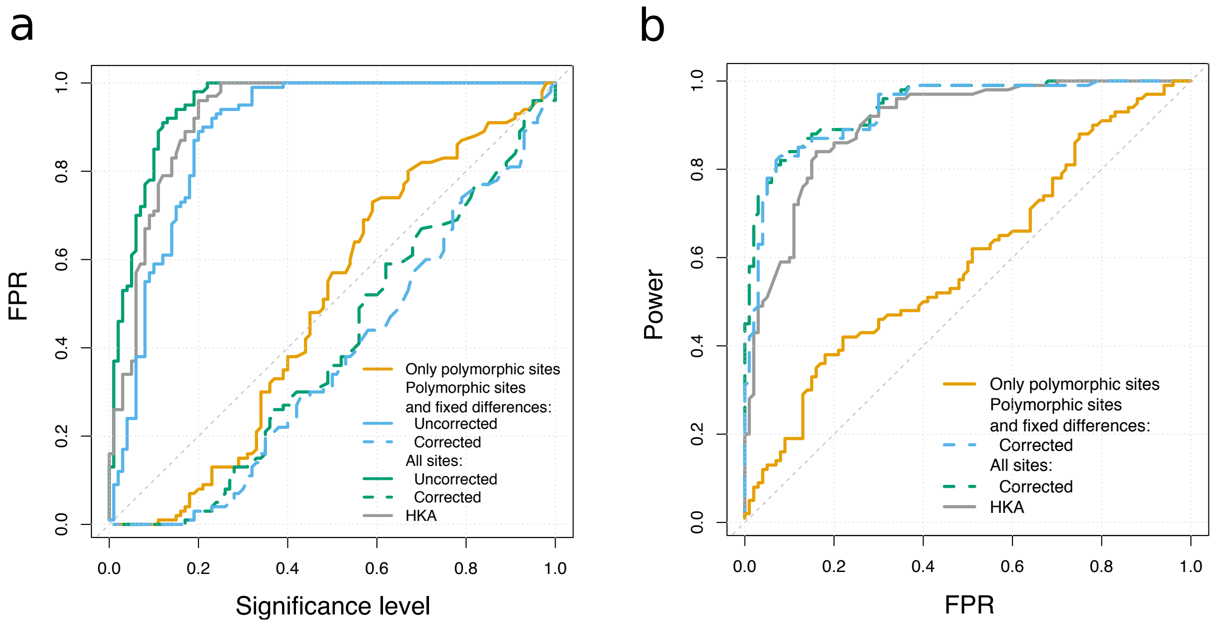

Figure S7. FPR and power under both background selection and a population bottleneck. The parameters of the bottleneck model are estimates for European humans (Lohmueller et al. 2011). a) The FPR is plotted against the nominal false positive rate (significance level). The nominal false positive rate is estimated from neutral simulations without background selection, but under the bottleneck model. b) The power of detecting a recently fixed selective sweep with $N_e s$ = 2000 as a function of the proportion of false positives.

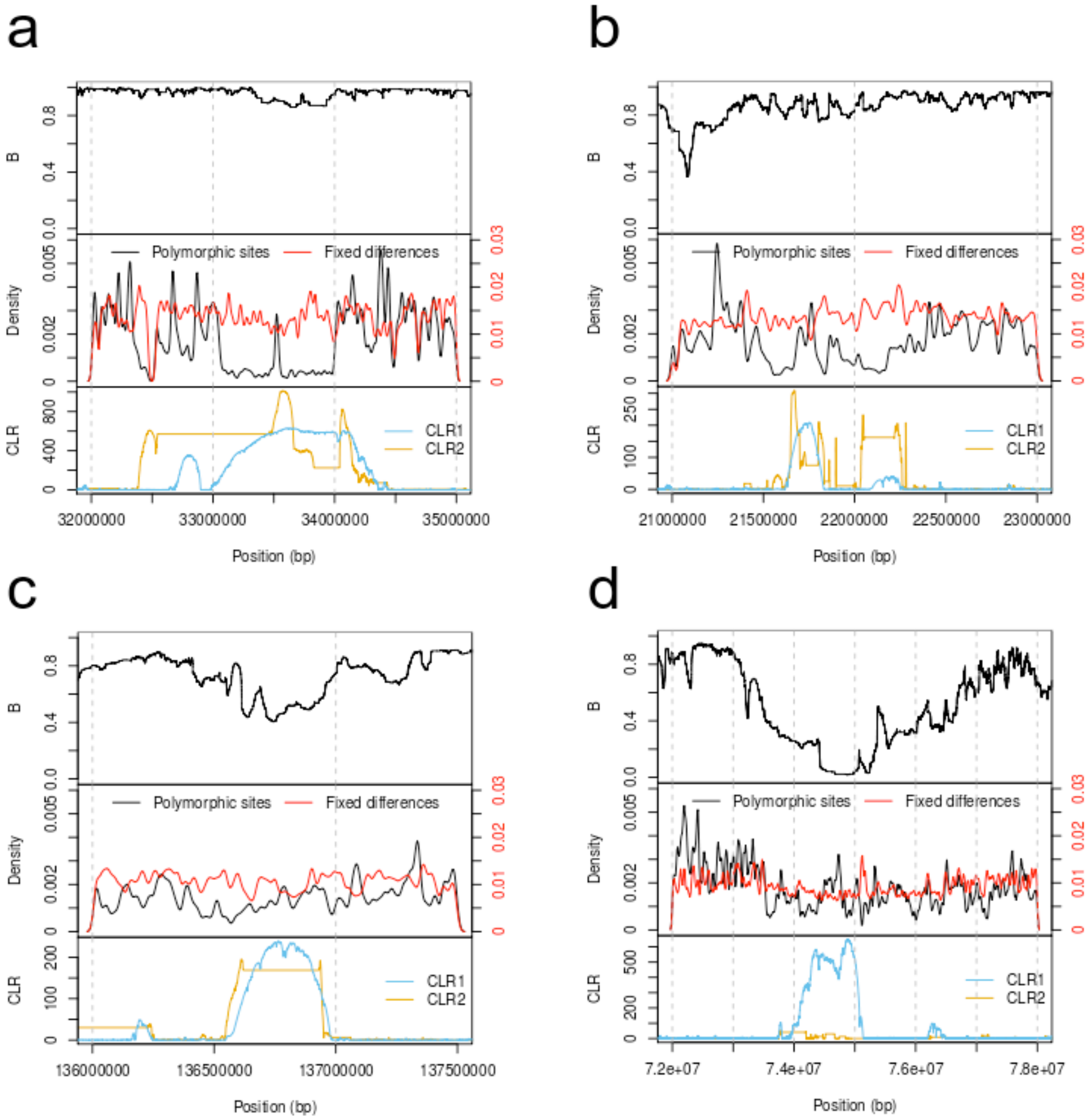

Figure S8. Examples from the human genome scan, running both the standard version of CLR using only polymorphic sites (CLR1), and our new version including fixed differences and the B-value map (CLR2). (a) to (d) show varying degrees of predicted background selection B from (McVicker et al. 2009), from almost no reduction in *B* at the specific region (a), a slight reduction in *B* in (b), a drop to about 50% in (c), and a reduction to almost zero levels in (d). Our new approach integrates the diversity to divergence ratio, the local allele frequency spectrum, and levels of predicted background selection for weighting in evidence for a selective sweep. Regions from (a) to (d): Chromosomes 4, 2, 6 and 10.

References

Ewing G, Hermisson J (2010) MSMS: A Coalescent Simulation Program Including Recombination, Demographic Structure, and Selection at a Single Locus. *Bioinformatics (Oxford, England)*, **26**, 2064–2065.

Lohmueller KE, Bustamante CD, Clark AG (2011) Detecting Directional Selection in the Presence of Recent Admixture in African-Americans. *Genetics*, **187**, 823–835.

McVicker G, Gordon D, Davis C, Green P (2009) Widespread Genomic Signatures of Natural Selection in Hominid Evolution. *PLoS Genet*, **5**, e1000471.
